# Bioclimatic comfort and solar responsive urban design in the traditional street texture of Diyarbakir’s Suriçi region

**DOI:** 10.1038/s41598-025-30582-5

**Published:** 2026-01-20

**Authors:** Kübra Suna Gider, Şefika Ergin, Hasan Yildizhan, Arman Ameen

**Affiliations:** 1 Department of Architecture, Faculty of Architecture, Dicle University, Diyarbakır, Turkey; 2 Energy Systems Engineering, Engineering Faculty, Adana Alparslan Türkeş Science and Technology University, 46278 Adana, Turkey; 3 Department of Building Engineering, Energy Systems and Sustainability Science, University of Gävle, 801 76 Gävle, Sweden

**Keywords:** Diyarbakir, Kabalti, Physiological equivalent temperature, Sky view factor, Thermal comfort, Climate sciences, Environmental sciences, Environmental social sciences

## Abstract

People in urban areas (such as streets, parks, semi-open and enclosed spaces) are exposed to varying microclimatic conditions. These conditions change depending on environmental characteristics and directly affect individuals’ bioclimatic comfort levels. The lack of climate-responsive urban planning exposes inhabitants to uncomfortable thermal stress. Establishing climate-sensitive thermal comfort conditions at the micro scale is therefore essential for creating more livable urban environments. In hot-arid climates, kabaltıs, roofed passages integrated into the street network, are among the spatial elements that influence pedestrian thermal comfort. However, there is limited knowledge in the literature regarding the thermal performance of these shaded structures, which provide both protection from solar radiation and shelter from rain and wind. This study aims to reveal the impact of kabaltıs, as traditional urban elements in hot-arid regions, on bioclimatic comfort, and to contribute to the development of climate-responsive urban design strategies. Due to the scarcity of research on the thermal performance of kabaltıs, the findings of this study provide new insights into climate-adaptive design solutions within traditional street networks and serve as a guide for urban planning practices. The research was conducted in the historical district of Diyarbakır Suriçi, focusing on six kabaltıs and their surrounding streets located in the Ziya Gökalp, Abdaldede, and Süleyman Nazif neighborhoods. At a total of 19 measurement points, air temperature, relative humidity, and wind speed were recorded over the course of one year. Using the RayMan Pro software, Physiological Equivalent Temperature (PET) values were calculated, and Sky View Factor (SVF) values were determined for comparative analysis. The results indicate that the studied streets and kabaltıs were exposed to varying degrees of heat and cold stress throughout the year. Shaded zones and kabaltıs exhibited lower air temperature and PET values compared to other points. In this hot-arid setting, the presence of covered, shaded areas was found to be effective in reducing solar exposure and lowering thermal stress during summer months. The measurements further revealed that urban geometry, particularly building height and street width, influenced solar radiation access and wind speed, thereby affecting PET values. In addition, no direct correlation was observed between SVF and PET, highlighting the need to consider other parameters when assessing bioclimatic comfort.

## Introduction

Human life is directly or indirectly influenced by environmental conditions. Among these, atmospheric events, key components of physical environmental factors, have significant impacts on both human health and daily life. As early as 2500 years ago, Hippocrates observed that regional climatic variations could affect human well-being. Historically, even before modern urban design practices were formalized, climatic conditions were considered critical for shaping built environments that support bioclimatic comfort. For instance, evidence suggests that as early as 65–68 BCE, studies in Rome explored the health impacts of climate and informed urban planning accordingly^1,2^. In urban areas, people encounter a variety of microclimates as they move through outdoor spaces such as streets, parks, and plazas. The structural characteristics of these spaces, including their dimensions and built density, can significantly influence levels of thermal stress experienced by users. For example, in hot summer climates, thermal stress can be mitigated by incorporating shaded zones and enhancing ventilation through wind flow. Conversely, in cold regions, wind protection becomes a design priority. Reducing thermal discomfort in outdoor environments is thus crucial for promoting the use of public spaces and enhancing user comfort^3^. Thermal comfort in open spaces directly affects the usage rate and duration of these areas. It encourages an increase in social, cultural and sporting activities by allowing people to spend more time outdoors, while also contributing to a reduction in energy consumption. This shows that it plays an important role in terms of both individual comfort and environmental sustainability^4–6^. Recent research increasingly focuses on the intersection of urban design and microclimatic conditions^7–13^. The American Society of Heating, Refrigerating and Air-Conditioning Engineers (ASHRAE) defines thermal comfort as a state of mind that reflects satisfaction with the thermal environment. This concept encompasses physical, physiological, and psychological dimensions. Thermal comfort arises when the human body achieves thermal equilibrium with its surroundings, and it is influenced by factors such as conduction, convection, radiation, and evaporative heat loss^14–16^. Accurate estimation of thermal sensation requires consideration of multiple variables, including air temperature, humidity, wind speed, mean radiant temperature, clothing insulation, activity levels, and individual characteristics^17^. The development of indices to assess thermal comfort zones gained momentum after the 1960s with technological advancements. Many of these indices were tailored to regional geographic and climatic conditions^18^. Among them, the Physiological Equivalent Temperature (PET) is one of the most widely adopted indices worldwide^19^. PET is based on the human body’s energy balance, specifically the principle that the total of heat produced and lost by the body should equal zero. This index allows for evaluating thermal comfort conditions by simulating indoor thermal conditions in an outdoor setting. It not only incorporates climatic elements but also accounts for physiological human responses. Expressed in degrees Celsius (°C), PET provides intuitive and internationally comparable results, facilitating its widespread use in environmental and urban studies^12,20–22^. The Sky View Factor (SVF), which has a decisive influence on the duration and intensity of exposure to sunlight, expresses the amount of sunlight received by an area as a percentage of the visible sky when viewed from a fixed point. Additionally, the Main radiant temperature (MRT) of an area is directly and strongly related to the SVF, regardless of whether the source of shade is trees or buildings. This relationship is critical for both evaluating thermal comfort and developing strategies to reduce the urban heat island effect^23,24^. Many cities located in hot and dry climates are built with deep, narrow streets that typically have low SVF values in order to reduce exposure to sunlight and increase thermal comfort. These types of street structures cause air temperatures to drop, especially in the summer months. Such designs are important for increasing comfort in urban life^25^.

Microclimatic conditions in urban areas are directly related to factors such as street geometry, orientation, shading, and vegetation, and these factors play a significant role in determining pedestrian thermal comfort. Studies in the literature demonstrate the effects of street geometry and orientation on PET (Physiological Equivalent Temperature) and other thermal comfort indicators. Ali-Toudert et al.^26^ demonstrated through simulations in Algeria that street aspect ratio (H/W) and orientation significantly alter PET values. Johansson et al.^25^ demonstrated through field measurements in Fez/Morocco that compact designs provide comfort through shading in the summer months, while more open streets that allow for sunbathing are needed in the winter months. Yılmaz et al.^27^ also noted in their study in Erzurum that sunny areas provide comfort in the winter and shaded areas in the summer. Simulations of open spaces in Ghardaia^28^ revealed that geometry, orientation, vegetation, and albedo are determinants of thermal comfort. Simulations of 64 scenario streets in Nanjing^8^ and optimization studies in Egypt^29^ also show that the size and orientation of street canyons directly affect daytime and nighttime thermal comfort.

Baghaeipoor et al.^30^ investigated the effect of SVF values on the microclimate. They determined that low SVF values provide shade and coolness in summer while blocking sunlight in winter, while high SVF values lead to overheating in summer. Analyses in other studies have shown that pedestrians prefer high comfort areas and that street trees and orientation have effects on PET^16,31,32^. Summer measurements in Tel Aviv assessed pedestrian thermal stress in sunny areas and revealed that tall buildings were effective in reducing pedestrian thermal stress. Simulation studies in Port Said investigated the effects of H/W, street orientation, and building typology on thermal comfort and found that linear N-S and NW–SE orientation with H/W = 2.5 was the optimal combination^33^. These studies reveal that street geometry, orientation, and SVF have significant effects on thermal comfort. The limited nature of measurements and analyses conducted in semi-open spaces such as kabaltı/abbara/sabat within the traditional urban fabric highlights the original contribution of this study in calculating thermal comfort using PET-based analyses in kabaltı and surrounding street canyons.

In traditional settlement structures, semi-open microclimate spaces known as “covered street passages” are known by different names across Anatolia and the Middle East. This typology, known as abbara in Mardin, kabaltı in Diyarbakır, Urfa, and Kilis, and sabat in North Africa, the Levant, and Iran, is also referred to as kab, kantarma, tetirbe, körü, köprü eğmesi, or geçit in various cities across Anatolia. Kabaltı/abbara, built to provide uninterrupted street passage, are constructed with cradle or cross vaults in some examples, and with wooden beam flat roofs in others. The upper floors of these passage spaces are generally used for specific functions such as rooms, sofas, or storage. These structures create comfortable spaces by providing shade within the streets^34^. Smail et al.^35^ have shown that covered streets improve the walking experience by reducing pedestrian fatigue through providing shade and airflow, contributing to the design of thermally comfortable walkable spaces. Younsi et al.^36^ revealed that traditional sabat structures and different street geometries in Tunisia are important in increasing outdoor thermal comfort. Büyükkırcalı et al.^37^ revealed that the kabaltı structures in the historic urban fabric of Şanlıurfa play an important role in both public and private spaces with their shading and protective functions.

Traditional housing areas are long-standing architectural forms shaped by regional climatic and geographic conditions. These structures typically reflect an adaptive harmony with the local climate and are designed to support occupant comfort. In Diyarbakir, Turkey, a region characterized by a hot and dry climate, traditional dwellings were specifically designed to mitigate the harsh environmental effects. Climatic factors have played a significant role in shaping the local architectural identity, guiding the spatial organization of homes and streets in the historical urban fabric. This study investigates bioclimatic comfort conditions in the traditional urban settlement of Diyarbakir Suriçi, focusing on streets of varying widths and the architectural element known as “*kabalti”*. Hereafter, the term “kabalti” will be used without quotation marks to refer to this specific structural feature, and kabaltis will denote its plural form.

This study aims to determine the advantages that kabaltı elements, which create shaded areas, provide against the adverse characteristics of the climate through a comparative PET analysis of these elements and the streets to which they are connected. For this purpose, the bioclimatic comfort conditions of the streets and street elements within the traditional settlement of Diyarbakır Suriçi were evaluated through on-site measurements. The quantitative findings of the study will shed light on the planning of similar shading elements and street geometries in a climate-responsive manner for new settlements. In addition, understanding the significance of such elements in traditional settlements will serve as a guide for conservation, renewal, and replanning studies.

## Methodology

### Study area

**Fig. 1:**
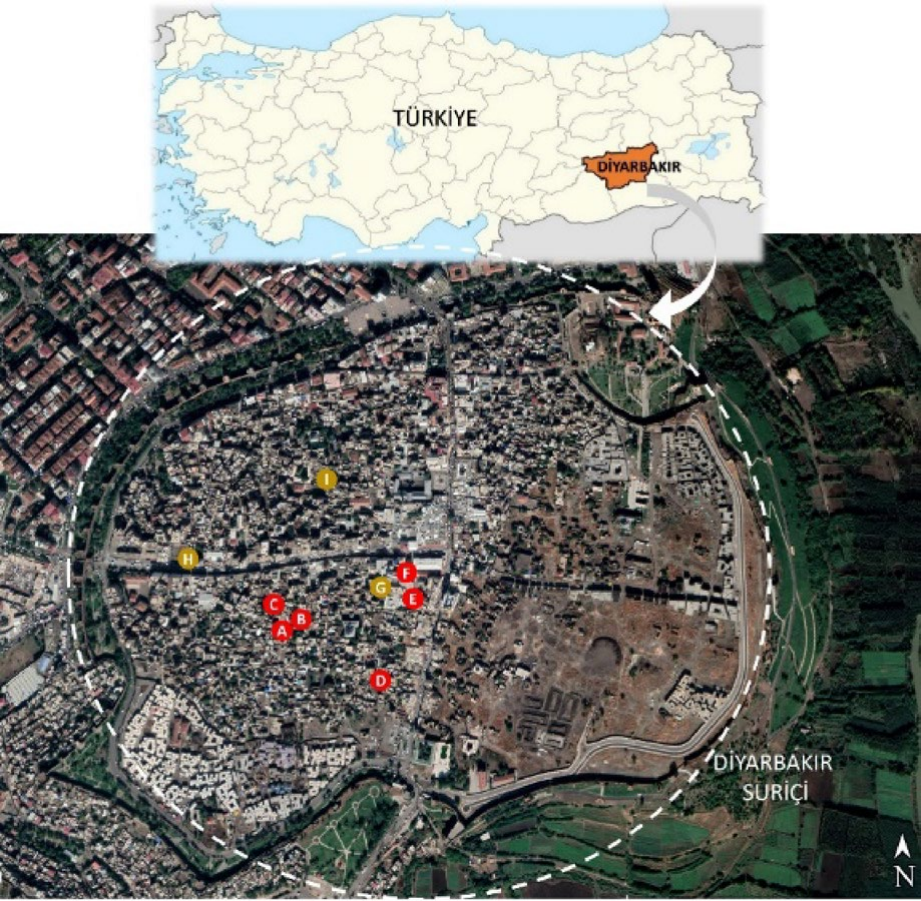
Location of Diyarbakir province on the map of Türkiye and measurement points in the Suriçi region (Measurement points were processed using the Power Point 2016 program on the image taken from^39^)^39,40^.

Diyarbakir Province, located in the central part of the Southeastern Anatolia Region of Turkey, lies at approximately 38° latitude and 40° longitude, as shown in Fig. 1. The city is situated to the east of the Karacadağ basalt plateau, on a basalt plain that rises 100 m above the Tigris Valley, at an elevation of 675 m above sea level. The first settlements in Diyarbakir Province began in the area known as the “inner castle,” which is enclosed by the city walls. Due to environmental factors, urban development continued for an extended period within these walls. This led to a compact urban fabric in the Suriçi region^38^.

**Fig. 2:**
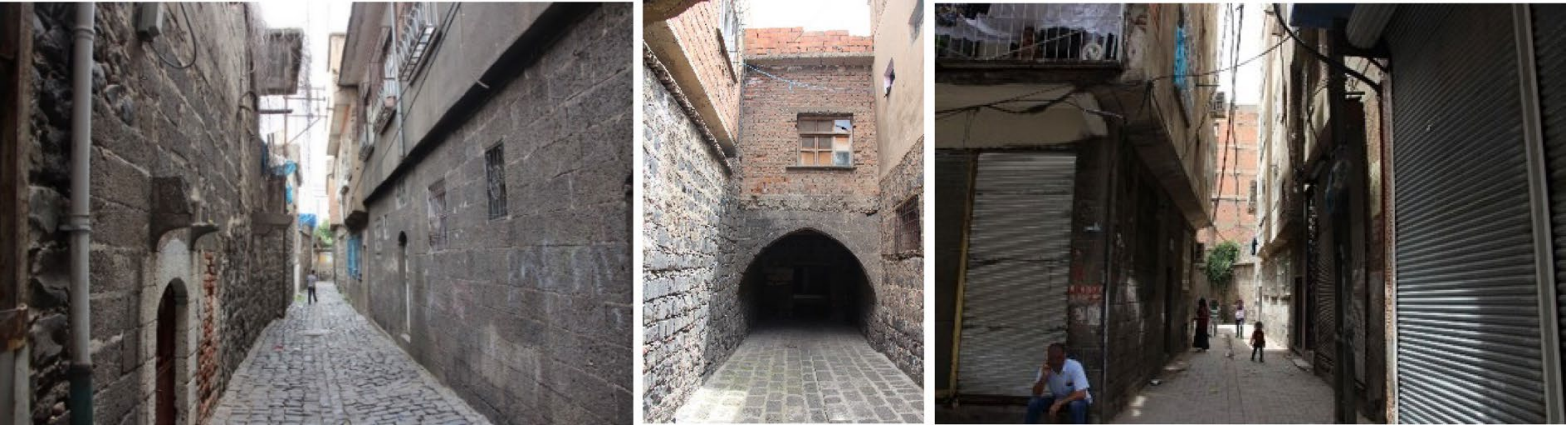
Representative photographic views of Diyarbakir’s street texture. Photographs were taken by the author [Kübra Suna Gider, June 2021] and are adapted from the author’s thesis^41^. All elements are published under a CC BY 4.0 license.

Within this settlement area, both low-rise courtyard buildings constructed from basalt and high-rise buildings made of brick, briquette, and similar materials coexist (Fig. 2). This contrast indicates that the traditional urban fabric of the settlement area has diminished over time.

Diyarbakir Suriçi traditional houses, which were created based on data obtained through long-term experiences, were designed with climate and socio-cultural factors in mind. These residences have created a balance between construction and the natural environment, creating comfortable spaces. The original street texture has been formed by the adjacent planning of the courtyard buildings formed in the region. Thanks to the high walls formed by the introverted space organization in the houses, quite shady and cool areas are formed on the streets in summer. In addition, most of the spaces in these houses are oriented towards the courtyard, and the number of spaces directed to the street is very few. With the expansion of the spaces facing the street in some residences, passages were formed in the lower parts of the rooms that overflowed the street. There are 9 kabaltis in Diyarbakir Suriçi region, and Fig. 1 shows the location of the existing kabaltis on the map of Suriçi region by marking them as A, B, C, D, E, F, G, H, and I. The layout, drawing, and photographic views as well as characteristics of designated kabaltis are presented in Table 1. The kabalti in the region varies in east–west and north–south orientations. Basalt stone was generally used as a building material, and bricks and briquettes were used in some. In addition, there are plastered walls of basalt stone in the area. The upper covers are made of wooden beamed flooring, vaults, or flat reinforced concrete floors. While two of the spaces above them are a place belonging to the mosque, the others are the place of residence.

**Table 1:**
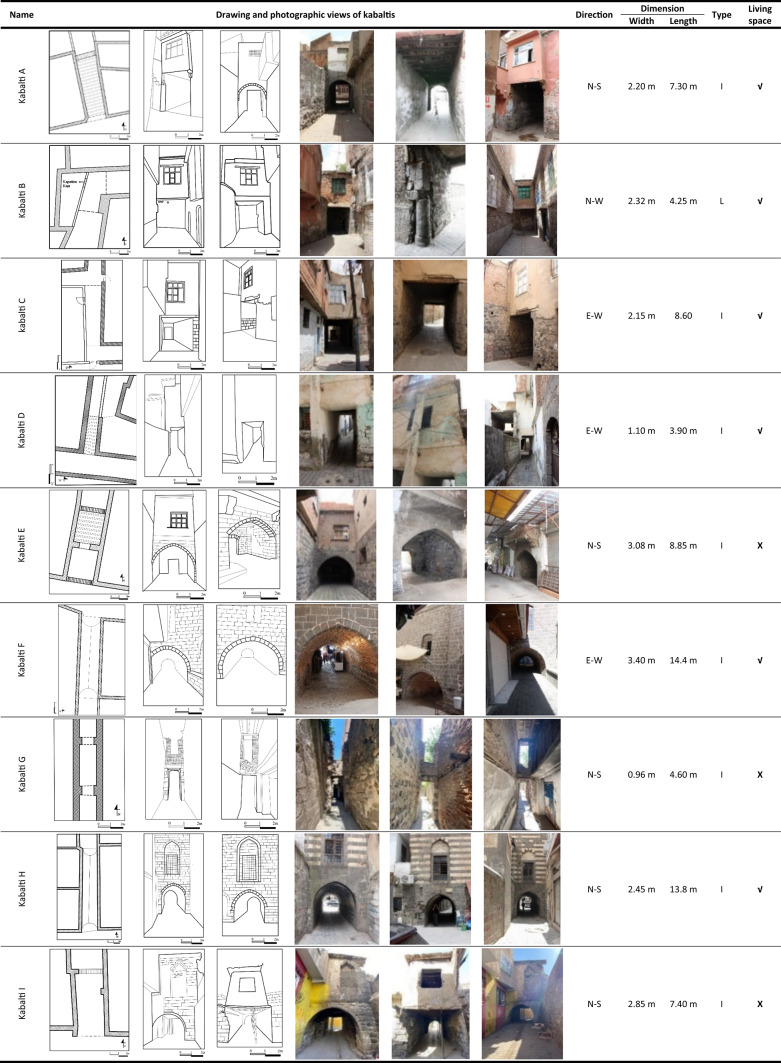
Layout, photograph and properties of kabaltis in Diyarbakir Suriçi region^41^.

### Climatic characteristics of the study area

Diyarbakir has a subtropical climate due to its location. It is seen as Csa according to Köppen Geiger climate classification. Some statistical metrics for the temperature, sunshine duration, precipitation values, etc. between 1929 and 2020 can be seen in Table 2^42^. The winter season in the region is very harsh, while the summer season is hot and dry. The average highest temperature value is 38.4 °C in July; The average lowest temperature value was measured in January with − 2.2 °C. The monthly average sunshine duration is the highest in July with 12.4 h. The average monthly precipitation amount is the highest in December with 72.2 mm.

**Table 2:**
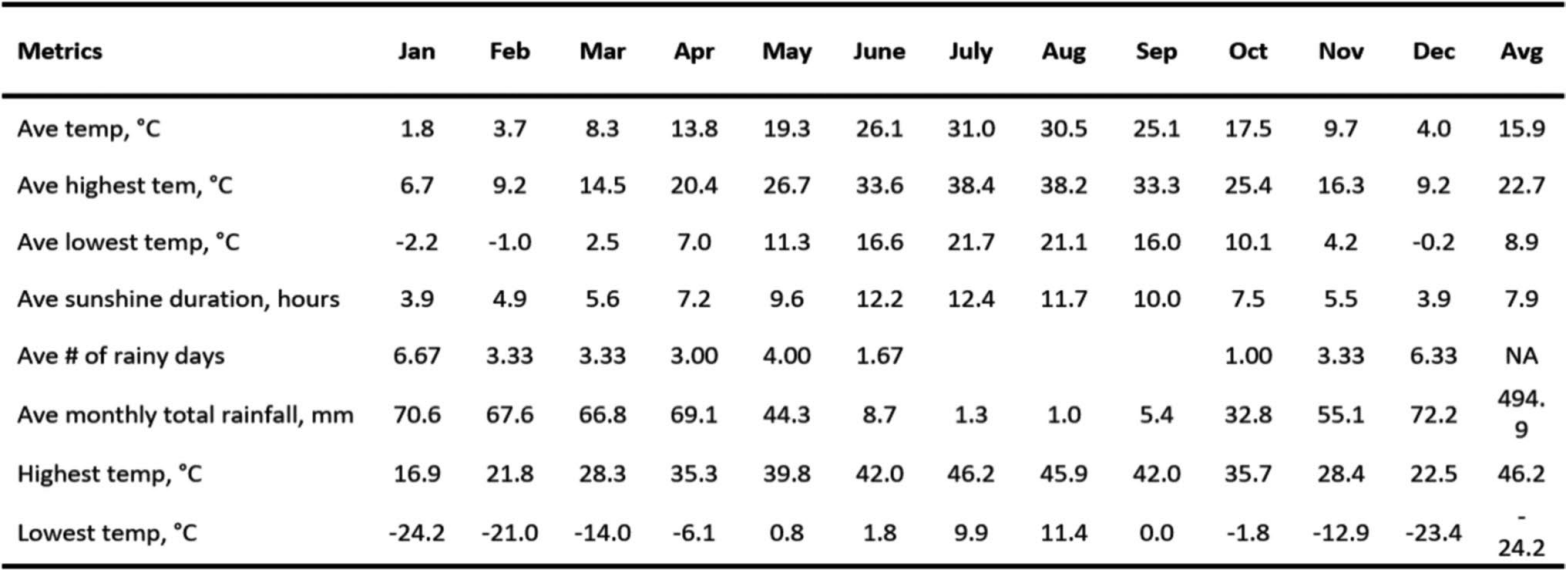
Some statistical metrics for Diyarbakir province determined from measurements taken between 1929 and 2020^42^.

The high amount of solar radiation received in Diyarbakir is due to its mid-latitude location and topographical flatness. The high amount of solar radiation received in Diyarbakir is due to its mid-latitude location and topographical flatness. In the summer season, when the days are long and the sun’s rays are steep, a daily sunshine duration of 12.4 h in July and solar radiation intensity reaching 3.05 MJ/m^2^ at noon in June are encountered. These results show that the comfort conditions are bad in the summer months^43^.

### Measurement and modeling process (RayMan model)

**Fig. 3:**
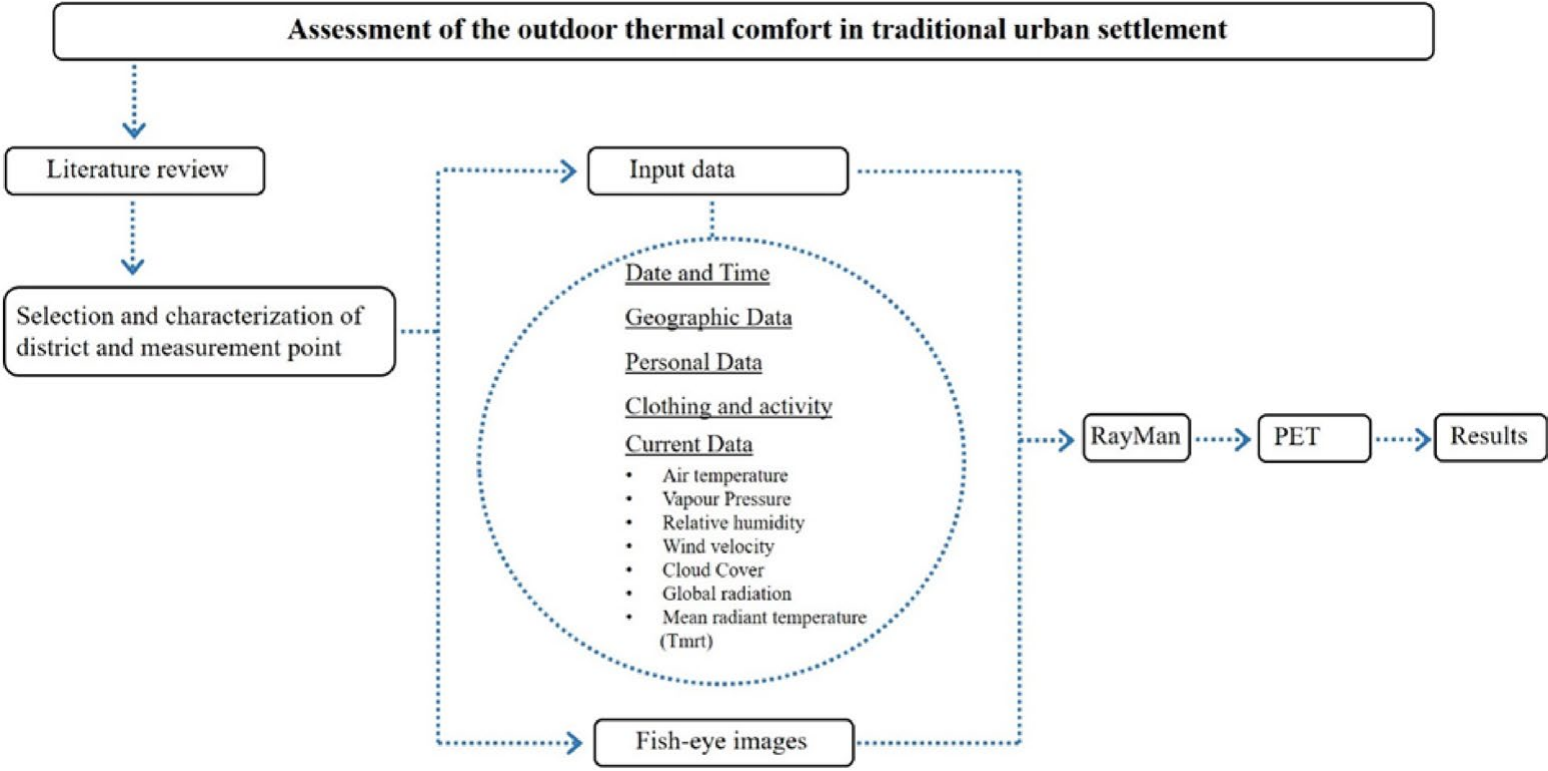
Flowchart of the methodology followed in this study.

There are a total of 9 kabaltis in the study area. Since the kabalti G and kabalti I are about to be demolished, and the kabalti H is far from the study area, these kabaltis were not included in the present study. A total of 25 points were designated from the kabaltis and the streets connected to kabaltis, and measurements were taken from these points. A-B-C-D-E-E1-F encodings indicate the points under the kabalti, while other encodings indicate the street points to which the kabalti is connected (see Table 1 and Fig. 1). Since the kabalti E is long, measurements were taken from both ends of the bottom of the kabalti (E, E1). The fact that the on-site measurement clock was close to each other in order not to deviate was the determining factor in the selection of the kabalti. The study was conducted according to the methodology outlined in Fig. 3. On-site measurements were made at the points selected for the study. Air temperature, humidity rates, wind speed values and SVF parameters were measured at the determined points. In order to calculate the SVF value, photographs were taken with a fisheye lens at a height of 1.5 m from the ground from the determined points. In the study, PET and SVF values as bioclimatic comfort index were calculated using the RayMan Pro program. Measurements in the study area were taken on the specified days and hours with a ± 5 min difference. In addition, a time difference occurs between taking measurements from each point and reaching another point, and the angle of incidence of the sun changes during this time. For this reason, the hours and dates entered into the calculation for each region in the RayMan Pro software may differ slightly. These hours; For Zone A, it is 09:10–14:10, for Zone B, it is 09:15–14:15, for Zone C, it is 09:25–14:25, for Zone D, it is 09:40–14:40, for Zone E, it is 09:50–14:50, for Zone F, it is 10:00–15:00 and the calculations were made according to the measurements at these hours. The measurements were taken at intervals of approximately 10 days between the beginning of June 2020 and the end of May 2021. The measurement hours are between 09:10 to 10:00 and 14:10 to 15:00. A total of 36 measurements were taken in a year.

**Fig. 4:**
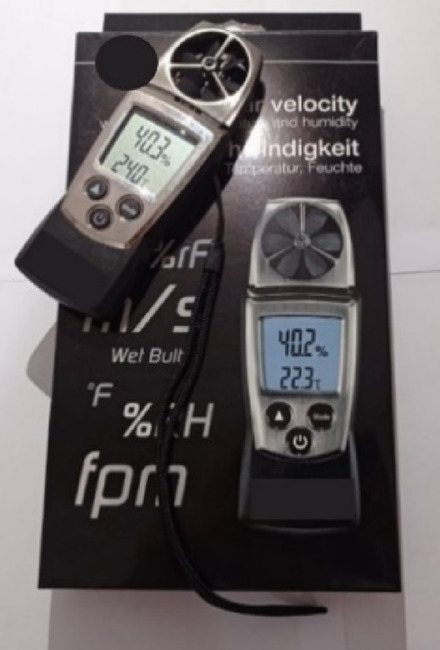
Testo 410-2 Propeller commercially-named anemometer employed in the measurements.

The Testo 410-2 Propeller commercially named anemometer, shown in Fig. 4, was used to measure the climatic data in the area, and a fisheye lens was used for the SVF values. Since the mean radiant temperature value could not be measured, it was neglected in the calculations. Instant PET value calculations were made for each region using climate data taken from the field.

According to the values calculated with the PET index, different thermal perception ranges were created in areas with different climate classifications. Some of the thermal perception ranges obtained as a result of the studies carried out according to the Köppen Geiger climate classification are given in Table 3 below^44^. As our study area has a Csa climate classification similar to the study conducted in Tel Aviv, Israel (Csa) by^45^, as shown in Table 3, their thermal sensitivity ranges were considered in the PET assessments.

**Table 3:** Thermal perception ranges of PET values for different climatic regions.

Termal comfort stress level	Central/western Europe^46^	Taiwan Sun Moon (Lake)^20^	Israel (Tel Aviv)^45^	China (Guangzhou)^47^	Turkiye (Konya)^44^
	Cfb	Cwa	Csa	Cfa	Bsk
Very cold	< 4	< 14	< 8	–	< (− 5.6)
Cold	4–8	14–18	8–12	–	(− 5.6)–6.2
Cool	8–13	18–22	12–15	< 11.3	6.2–17.9
Slightly cold	13–18	22–26	15–19	11.3–19.2	–
Comfortable	18–23	26–30	19–26	19.2–24.6	17.9–29.7
Slightly warm	23–29	30–34	26–28	24.6–29.1	–
Warm	29–35	34–38	28–34	29.1–36.3	29.7–41.5
Hot	35–41	38–42	34–40	36.3–53.6	41.5–53.3
Very hot	> 41	> 42	> 40	> 53.6	> 53.3

## Results and discussion

Some of the buildings in the study area are high-rise and reinforced concrete, while some are traditional buildings with courtyards. The streets do not have a uniform layout, as the different building types change the heights of the buildings. In addition, the width and orientation of the measured streets are variable. These characteristic differences cause different SVF values on the street axes. In addition, Table 4 shows that although the characteristics of some streets are different (A1-C1, B2-B3, D1-D2-D3), their SVF values are similar. Since the effect of afforestation on the streets is minimal, the building heights were decisive in the SVF value. Temperature, humidity, wind speed data and calculated PET values measured for a year are expressed in graphics and tables and evaluated under separate headings. The locations, sections and fisheye photographs of the measurement points are shown in Table 4.

**Table 4:**
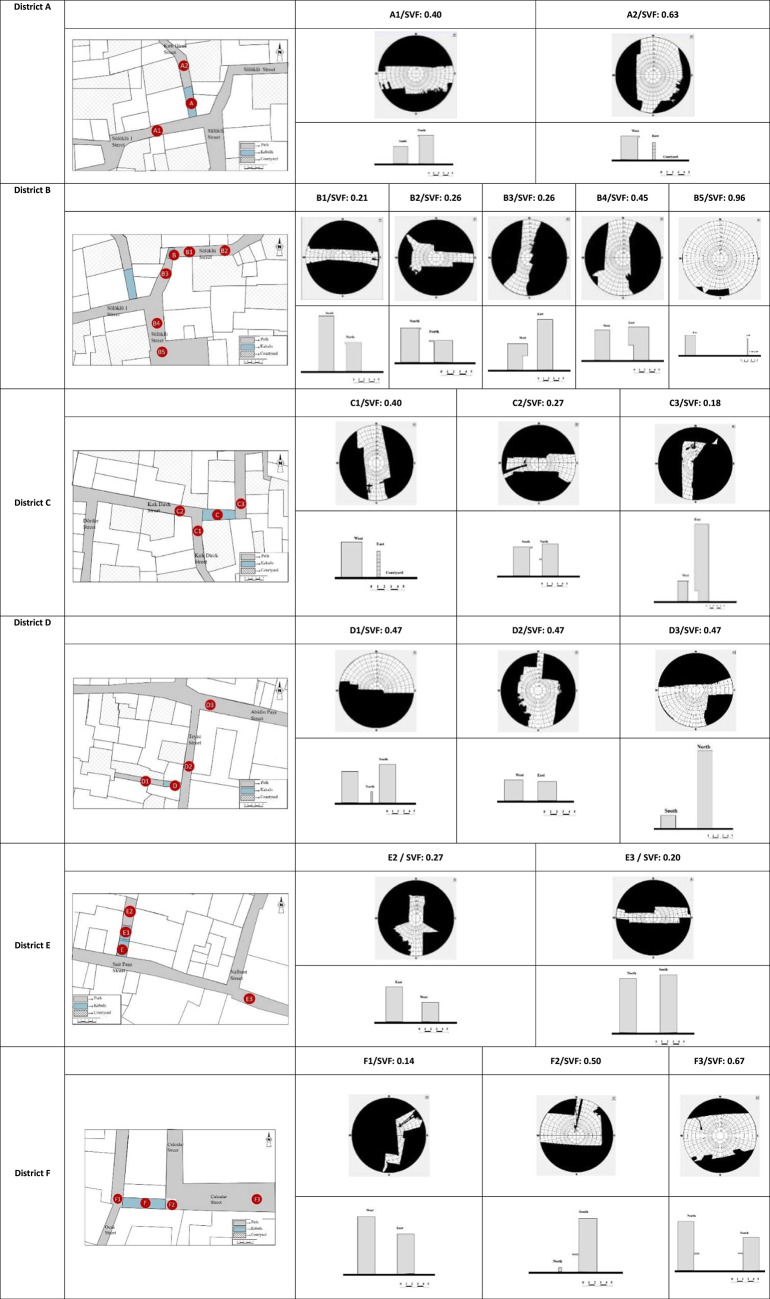
The properties of the measurement points. The map, showing a selected portion of the study areas, was derived from .dwg files provided by the municipality (used with permission). Measurement points (A–E), fisheye photographs, and field measurements were developed and collected by the authors. This table was adapted from the author’s thesis^41^”.

### Temperature

**Fig. 5:**
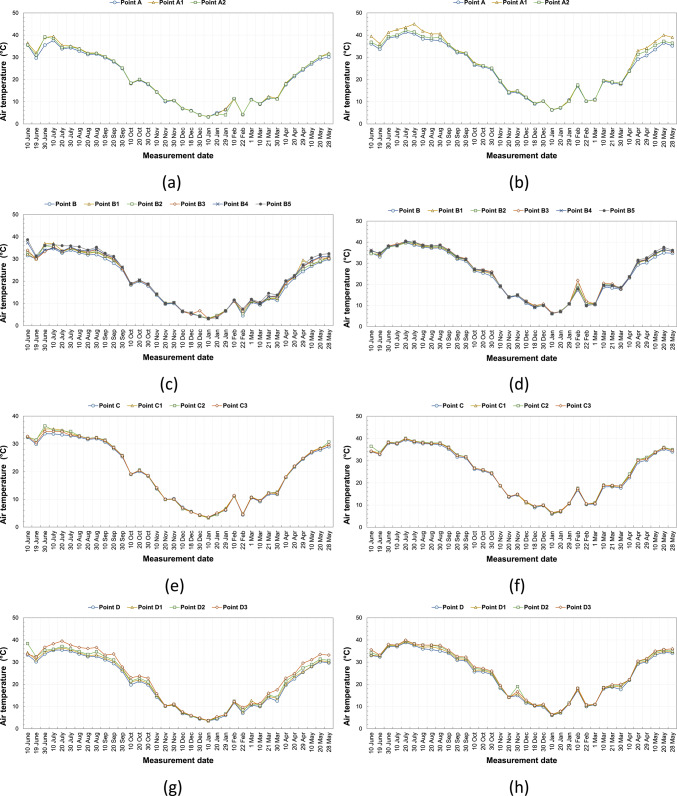

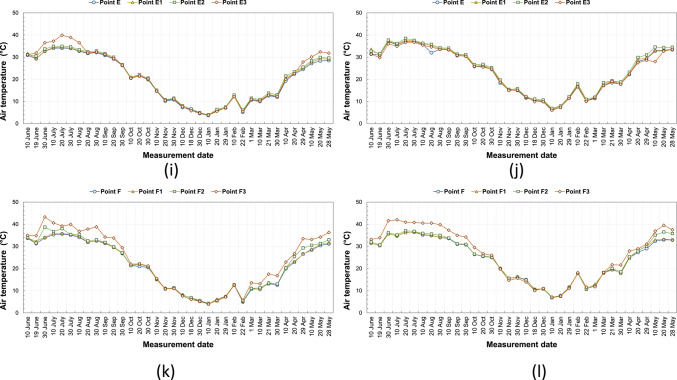
The temperature results measured between June 2020 and May 2021 periods taken at (**a**) 09:10—kabalti A, (**b**) 14:10—kabalti A, (**c**) 09:15—kabalti B, (**d**) 14:15—kabalti B, (**e**) 09:25—kabalti C, (**f**) 14:25—kabalti C, (**g**) 09:40—kabalti D, (**h**) 14:40—kabalti D, (**i**) 09:50—kabalti E, (**j**) 14:50—kabalti E, (**k**) 10:00—kabalti F, and (**l**) 15:00—kabalti F.

**Fig. 6:**
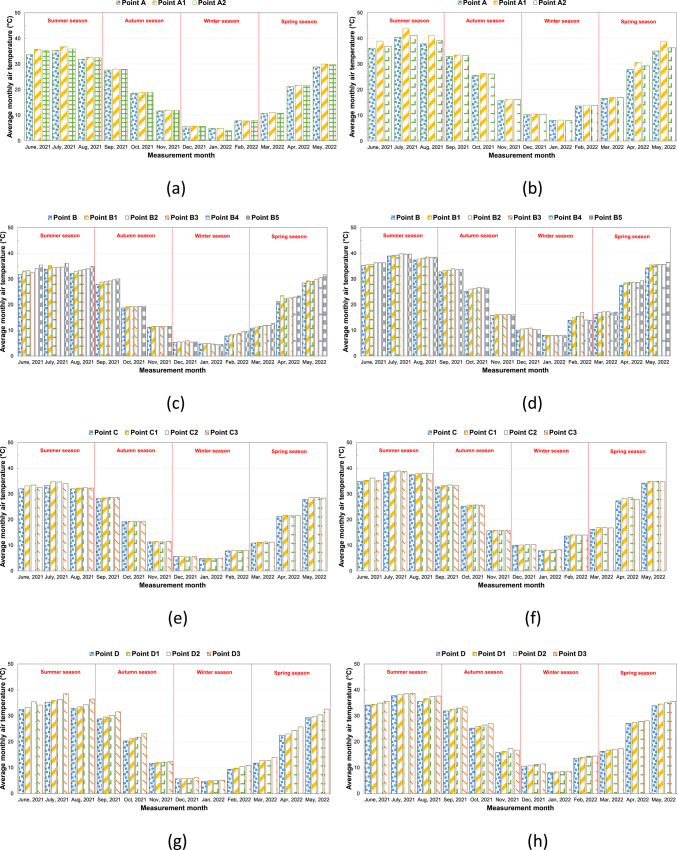

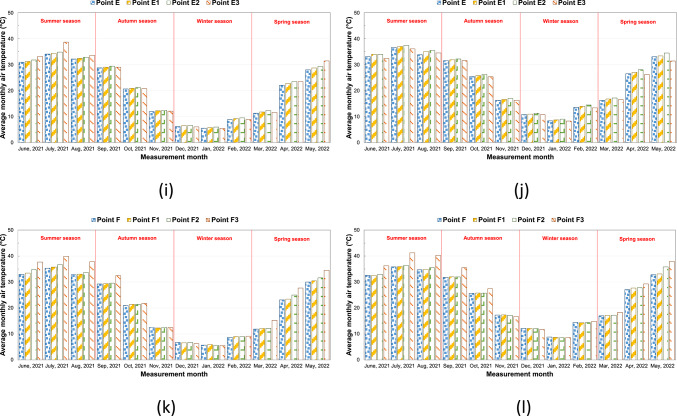
Average monthly air temperature versus measurement month for (**a**) 09:10—kabalti A, (**b**) 14:10—kabalti A, (**c**) 09:15—kabalti B, (**d**) 14:15—kabalti B, (**e**) 09:25—kabalti C, (**f**) 14:25—kabalti C, (**g**) 09:40—kabalti D, (**h**) 14:40—kabalti D, (**i**) 09:50—kabalti E, (**j**) 14:50—kabalti E, (**k**) 10:00—kabalti F, and (**l**) 15:00—kabalti F.

The air temperature values measured between 10/06/2020 and 28/05/2021 at kabaltis A, B, C, D, E, and F at 9.00 and 14.00 are shown in Fig. 5a–l, and average monthly temperature values are shown in Fig. 6a–l, respectively. According to the measurements in the morning hours in a region throughout the year, the highest air temperature is at A1 point with a value of 39.5 °C, and the lowest air temperature is at the A and A2 points with a value of 3.2 °C. According to the measurements in the afternoon, the highest air temperature in the A region is at the A1 point with a value of 45 °C, and the lowest air temperature is 6.3 °C at the A and A2 points. Point A1 is east–west oriented, and since it is exposed to a longer and more intense solar radiation intensity than point A2 from sunrise to sunset, the temperature value here is higher than other points. The fact that the SVF value of the A1 point is lower than the A2 point did not cause a significant change in the effect of the orientation factor (Fig. 5a, b). In winter and autumn, the afternoon temperature values of points A, A1 and A2 are very close to each other. In summer and spring, the highest monthly average temperature was recorded at point A1 (Fig. 6a, b). In the morning measurements in the B region, the highest air temperature is 38.7 °C at the B5 point, and the lowest air temperature is 3.1 °C at the B2, B3, and B4 points. In the afternoon, the highest air temperature was recorded at the B5 point with a value of 40.6 °C, and the lowest air temperature was recorded at the B, B1, B2, and B5 points with a value of 6.1 °C. The fact that the temperature value of the B5 point is higher than the other points in the morning and afternoon measurements is related to the higher SVF value and the street geometry. The temperature difference between morning and afternoon at point B varied between 3 and 6 °C (Fig. 5c, d). According to the average monthly data of the morning temperature, it has been observed that point B5 generally has the highest value of the temperature during the summer and spring periods. This situation was caused by point B5 being exposed to direct sunlight during the morning hours. Point B is usually the point with the lowest temperature value according to the monthly average temperatures (Fig. 6c, d). In the morning measurements in the C region, the highest air temperature is 36.5 °C at the C2 point, and the lowest air temperature is 3.3 °C at the C1 point. In the afternoon measurements, the highest air temperature in the C region is 40.1 °C at the C2 point, and the lowest air temperature is 6.0 °C at the C point. The temperature value at this point is higher because the SVF value of the C2 point is higher than the other points and because it is east–west oriented as well (Fig. 5e, f). The monthly average temperature values of all points in C are very close to each other in winter and autumn periods (Fig. 6e, f). In the morning measurements in the D region, the highest air temperature is at D3 point with a value of 39.5 °C, and the lowest air temperature is at D1 point with a value of 3.5 °C. In the afternoon, the highest air temperature with a value of 39.9 °C is at point D3, and the lowest air temperature is at point D with a value of 6 °C. The D3 point is generally higher up to about 3.0 °C than the D, D1, and D2 points. This difference is related to the orientation of the street. The values of D and D1 points are very close to each other. This situation is due to the fact that the D1 point is shaded during the day, depending on its geometry. Although D1, D2, and D3 points have the same SVF values, they take different temperature values depending on the combination of parameters such as urban geometry, wind, and orientation (Fig. 5g, h). Point D3 usually has the highest average monthly temperature in the morning hours, with the exception of the winter months. Point D usually has the lowest temperature value according to monthly average temperatures (Fig. 6g, h). In the morning measurements in the E region, the highest air temperature is at the E3 point with a value of 39.9 °C, and the lowest air temperature is at the E point with a value of 3.7 °C. In the afternoon measurements, the highest air temperature in the E region is at the E2 point with a value of 38.5 °C, and the lowest air temperature is at the E3 point with a value of 6.0 °C. The temperature differences measured in the morning and afternoon at points E and E1 are low. This situation is related to street geometry and orientation (Fig. 5i, j). The highest average monthly temperature in the morning hours is at point E3 during the summer period. E2 usually has the highest midday temperature throughout the whole period. (Fig. 6i, j). In the F region, the highest air temperature in the morning measurements is 43.3 °C at the F3 point, and the lowest air temperature is 4.1 °C at the F point. According to the measurements in the afternoon, the highest air temperature in the F region is 42 °C at the F3 point, and the lowest air temperature is 6.8 °C at the F2 point. It has been determined that the temperature values of the F3 point are up to about 10.0 °C higher than the other two points in some of the seasonal transitions and in the summer months, and in the winter months, all four points have values very close to each other. This difference in summer months is related to the high SVF value of the F3 point and its exposure to excessive solar radiation due to the street geometry (Fig. 5k, l). During the winter period, the monthly average temperature values of all points in F are close to each other. In other periods of the year, the monthly average temperature value of point F3 is significantly higher than the other points (Fig. 6k, l).

By examining the hourly temperature data from meteorology, it was determined that the highest temperatures were observed on 20/07/2020 during the year. In the measurements made on this date, it was observed that the temperatures of the kabalti points were lower than the outdoor temperatures. Accordingly, the use of kabalti in the streets is an architectural planning element that provides an advantage in the hot period of the year as can be seen in Table 5.

**Table 5:** Temperature values taken from the kabalti points and meteorological records on July 20, 2020 (in °C).

		09.00 am	02.00 pm
Meteorological data		40.8	41.4
Measurements from kabaltis	A	33.9	41.3
	B	32.7	39.8
	C	33.3	39.4
	D	35.5	38.9
	E	34.1	37.1
	E1	34.3	37.7
	F	35.6	36.4

**Fig. 7:**
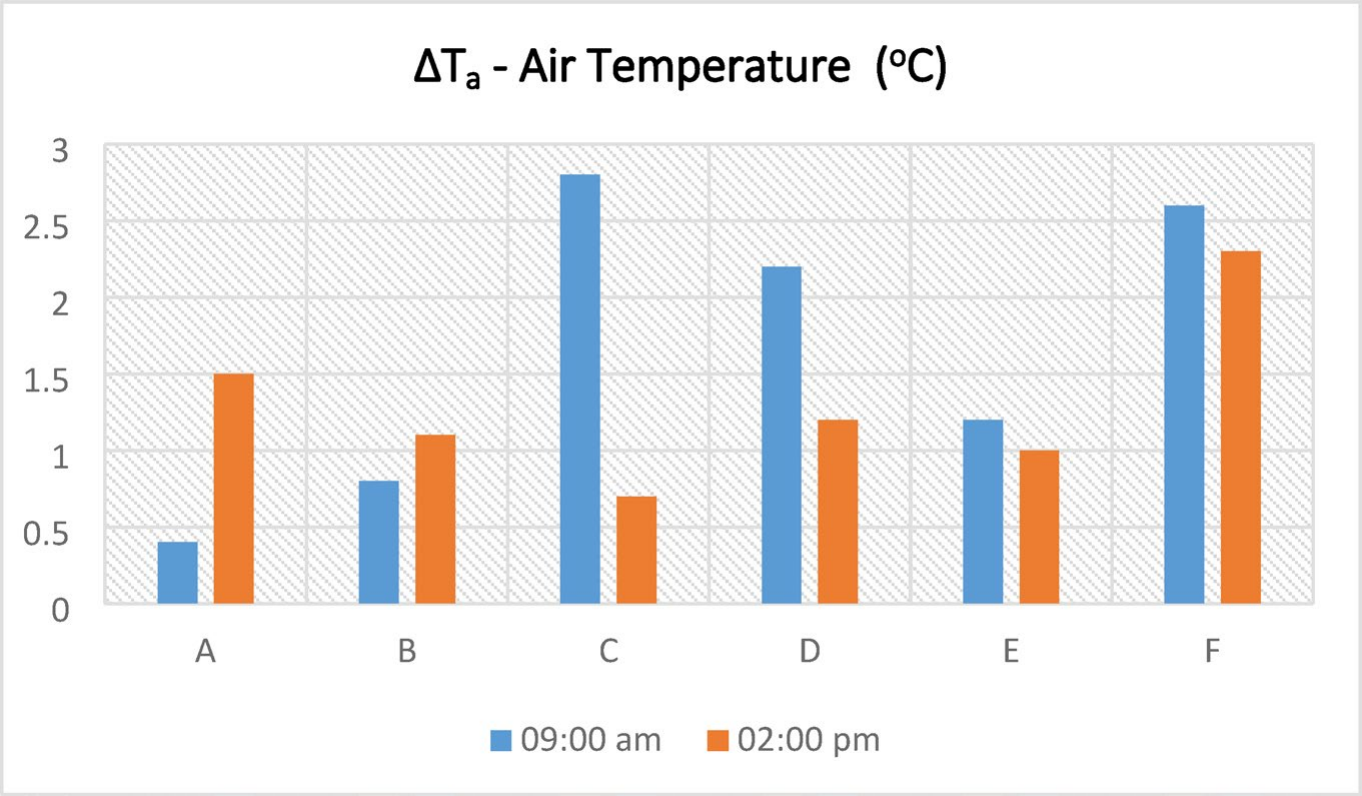
According to annual average values ΔT_a_ (T_max_ − T_kabalti_).

The ΔT_a_ results, calculated from the temperature data in Table 6, indicated that, in regions A, B, C, D, and E, the difference between the air temperature beneath the kabaltıs and the maximum air temperature varied up to 3 °C during the morning and noon hours. In contrast, the highest differences were observed as 2.8 °C in region C in the morning and 2.3 °C in region F at noon. These findings, which are directly supported by the values in Table 6, suggest that kabaltıs can play a more effective cooling role depending on their location and surrounding environmental conditions. Furthermore, these results demonstrate that kabaltıs significantly influence PET values through parameters such as radiation and shading (Table 6; Fig. 7).

**Table 6:** Annual average temperature values taken from the kabalti points (in ^o^C).

Time	Points					
	A	A1	A2			
09:00 a.m	19.8	20.4	20.2			
02:00 p.m	25.1	26.6	25.7			

Time	Points					
	B	B1	B2	B3	B4	B5
09:00 a.m	19.5	20.3	20.2	20.4	20.7	21.3
02:00 p.m	24.6	25.2	25.4	25.7	25.3	25.5

Time	Points					
	C	C1	C2	C3		
09:00 a.m	33.7	35.4	36.5	34.5		
02:00 p.m	39.4	39.8	40.1	39.9		

Time	Points					
	D	D1	D2	D3		
09:00 a.m	20.4	21	21.6	22.6		
02:00 p.m	24.2	24.7	25.2	25.4		

Time	Points					
	E	E1	E2	E3		
09:00 a.m	20	20.4	20.8	21.2		
02:00 p.m	23.8	24.3	24.8	23.6		

Time	Points					
	F	F1	F2	F3		
09:00 a.m	20.9	21.1	21.6	23.5		
02:00 p.m	24.2	24.3	24.6	26.5

### Wind

**Fig. 8:**
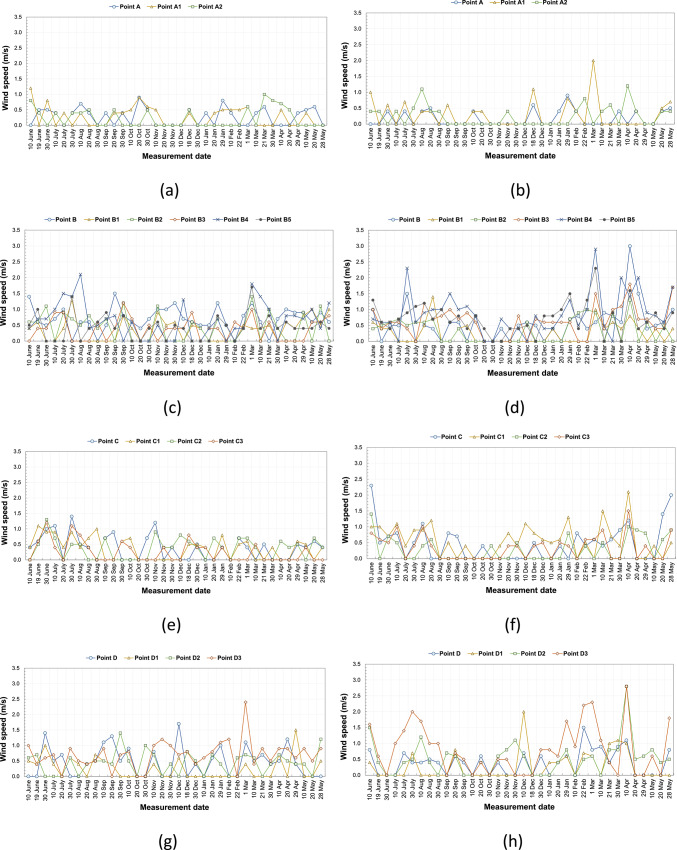

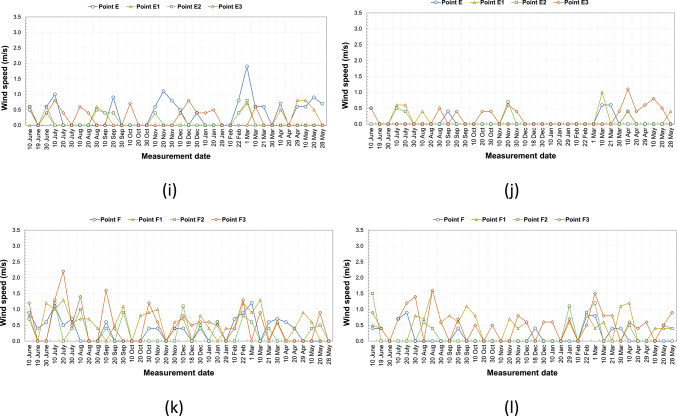
The wind speed results measured between June 2020 and May 2021 periods taken at (**a**) 09:10—kabalti A, (**b**) 14:10—kabalti A, (**c**) 09:15—kabalti B, (**d**) 14:15—kabalti B, (**e**) 09:25—kabalti C, (**f**) 14:25—kabalti C, (**g**) 09:40—kabalti D, (**h**) 14:40—kabalti D, (**i**) 09:50—kabalti E, (**j**) 14:50—kabalti E, (**k**) 10:00—kabalti F, and (**l**) 15:00—kabalti F.

Parameters such as SVF values, orientations, and widths of the streets are also effective in the wind speed in the measurements. Each of these parameters is not a factor on its own. Their different combinations with each other change the wind speed of the environment. The values of wind speeds measured in the study area are shown in Fig. 8a–l. There may be instantaneous changes in the wind speed affecting the region within approximately one hour of completion of the measurements. In this case, the wind speeds at the points also differ. The instantaneous wind speed change can also change in a short time interval in the same measurement region. This situation is seen in wind speed differences measured at the same time periods at the D1, D2, and D3 points with the same SVF values (SVF: 0.47). The highest difference in morning measurements between these three points was 2.0 m/s in March, and the highest difference in afternoon measurements was 1.8 m/s in April. The opposite is also possible. It was observed that the streets with different SVF values also had similar wind speeds in the measurements during the same time. F1 (SVF: 0.14) and F3 (SVF: 0.67) points are examples of this situation. It was observed that similar values were measured on some days of February, March, May, June, October, and November in the morning measurements at these two points and on some days of January, February, June, August, October, November, and December in the noon measurements.

In the quarterly evaluation, the lowest wind speed is between 0 and 0.1 m/s at points other than kabalti. The highest wind speed was measured at B4 with 1.4 m/s in spring, at the B4 point with 1.2 m/s in autumn, at the D3 point with 1.4 m/s in summer, and at the D3 point with 1.8 m/s in winter. The annual average wind speed measurements of all points are shown in Table 7. Accordingly, the highest value is 0.7 m/s in the morning and 0.8 m/s at noon. The wind speed values in the study area are in the range of calm, light air, and light breeze definitions according to the Beaufort (Bofor) Wind Scale^48^. According to these results shown in Fig. 8a–l, it can be said that not all of the points in the measurement area are exposed to very strong winds.

**Table 7:** Annual average wind speed values taken from the kabalti points (in m/s).

Time	Points					
	A	A1	A2			
09:00 a.m	0.2	0.2	0.2			
02:00 p.m	0.1	0.2	0.2			

Time	Points					
	B	B1	B2	B3	B4	B5
09:00 a.m	0.7	0.3	0.4	0.3	0.6	0.4
02:00 p.m	0.5	0.2	0.3	0.6	0.8	0.7

Time	Points					
	C	C1	C2	C3		
09:00 a.m	0.3	0.3	0.3	0.2		
02:00 p.m	0.4	0.5	0.2	0.3		

Time	Points					
	D	D1	D2	D3		
09:00 a.m	0.4	0.2	0.4	0.6		
02:00 p.m	0.3	0.2	0.5	0.8		

Time	Points					
	E	E1	E2	E3		
09:00 a.m	0.3	0.1	0.1	0.1		
02:00 p.m	0.06	0.09	0.04	0.1		

Time	Points					
	F	F1	F2	F3		
09:00 a.m	0.3	0.5	0.2	0.4		
02:00 p.m	0.1	0.4	0.1	0.5		

**Fig. 9:**
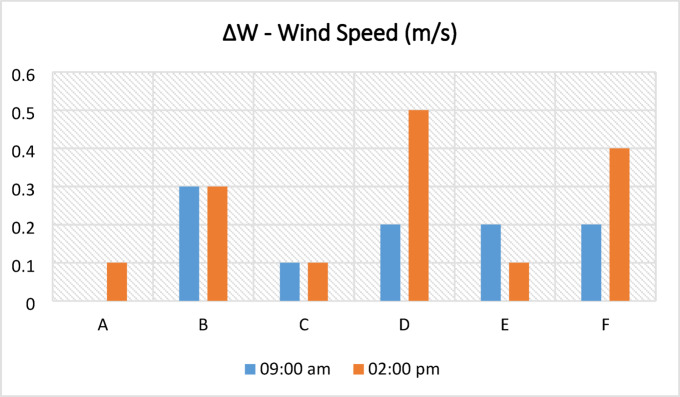
According to annual average values ΔW (W_maksimum_ −  W_kabalti_).

According to the morning measurements, the Δwind speed (ΔW) calculated based on annual averages ranged between 0 and 0.2 m/s, while at noon it varied between 0.1 and 0.5 m/s. In general, wind speeds were higher in open areas and lower under kabaltıs and in narrow streets. At noon, particularly at points B4 and D3, wind speeds reached 0.8 m/s, whereas at the E and E1 kabaltıs, wind speeds dropped to nearly 0 m/s. These findings indicate that kabaltıs and narrow street configurations restrict airflows, while wind movement is more pronounced in open areas (Fig. 9).

### Humidity

**Fig. 10:**
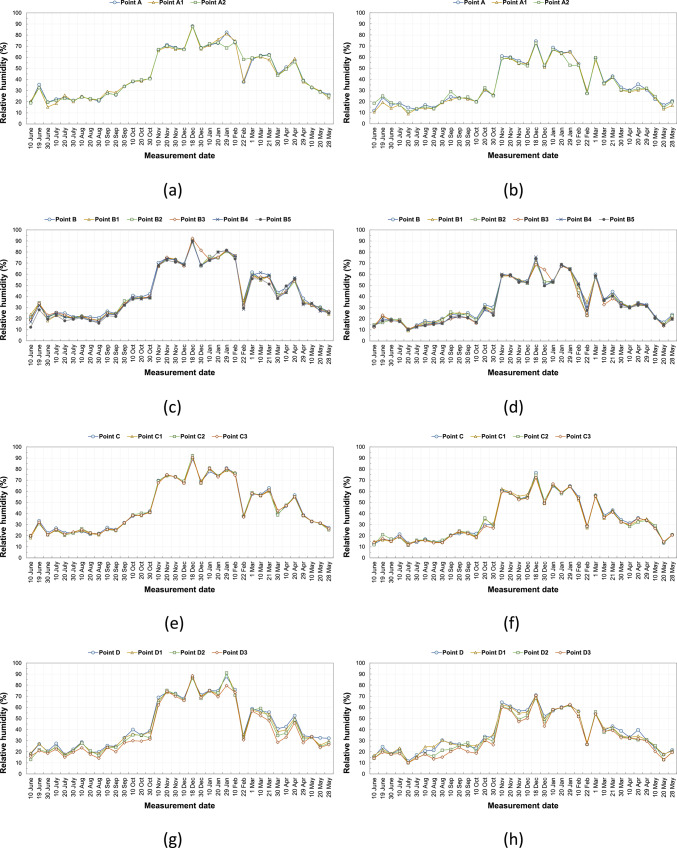

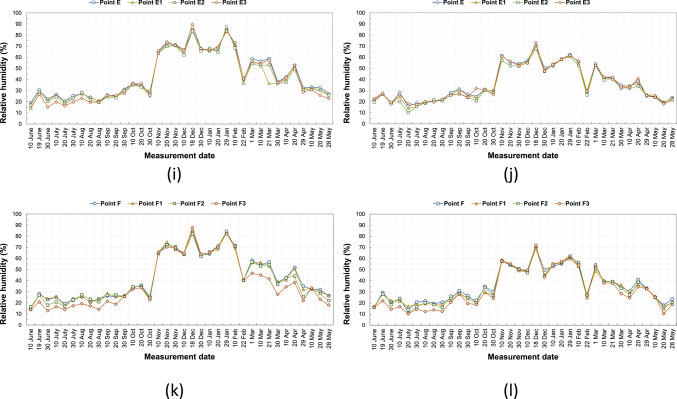
The relative humidity results measured between June 2020 and May 2021 periods taken at (**a**) 09:10—kabalti A, (**b**) 14:10—kabalti A, (**c**) 09:15—kabalti B, (**d**) 14:15—kabalti B, (**e**) 09:25—kabalti C, (**f**) 14:25—kabalti C, (**g**) 09:40—kabalti D, (**h**) 14:40—kabalti D, (**i**) 09:50—kabalti E, (**j**) 14:50—kabalti E, (**k**) 10:00—kabalti F, and (**l**) 15:00—kabalti F.

There are buildings with and without courtyards around the measurement points. The ornamental pools in the courtyards of some buildings and the water accumulating in the pores due to the wetting of the basalt stone on the ground floor affect the humidity of the environment and change the microclimatic feature of the region. Each measurement point has different structural characteristics and does not contain these elements that affect the microclimatic feature. For this reason, the humidity rates at the measurement points varied. Figure 10a–l shows the humidity values measured in the area on the specified hours and days. Humidity values measured at all points in the same duration are generally close to each other. According to the data measured, the humidity values in the morning are generally higher than in the afternoon. The annual average humidity shown in Table 8 were found to be within the range considered comfortable in the morning hours and below the comfort range in the afternoon. In the morning, the average lowest humidity is 38.4% at the F3 point, and the highest humidity is 46% at the B and C points. In the afternoon, the average lowest humidity is 32.1% at the F3 point, and the highest humidity is 36.6% at the D point (Table 8). In addition, the relationship between SVF values in the streets and humidity measurements was examined, and it was seen that SVF values alone did not affect humidity measurements.

**Table 8:** Annual average humidity values taken from the kabaltı points (in %).

Time	Points					
	A	A1	A2			
09:00 a.m	45.7	45.4	45.6			
02:00 p.m	35.0	33.6	34.4			

Time	Points					
	B	B1	B2	B3	B4	B5
09:00 a.m	46.0	45.0	44.9	45.2	44.7	43.5
02:00 p.m	34.9	34.5	33.7	33.1	33.6	33.2

Time	Points					
	C	C1	C2	C3		
09:00 a.m	46.0	45.7	45.8	45.8		
02:00 p.m	34.6	34.4	34.4	33.9		

Time	Points					
	D	D1	D2	D3		
09:00 a.m	45.2	44.0	42.8	40.7		
02:00 p.m	36.6	36.3	34.1	32.7		

Time	Points					
	E	E1	E2	E3		
09:00 a.m	44.0	42.3	42.1	42.4		
02:00 p.m	36.2	35.0	34.2	35.9		

Time	Points					
	F	F1	F2	F3		
09:00 a.m	43.0	42.7	41.9	38.4		
02:00 p.m	35.3	34.1	34.4	32.1		

**Fig. 11:**
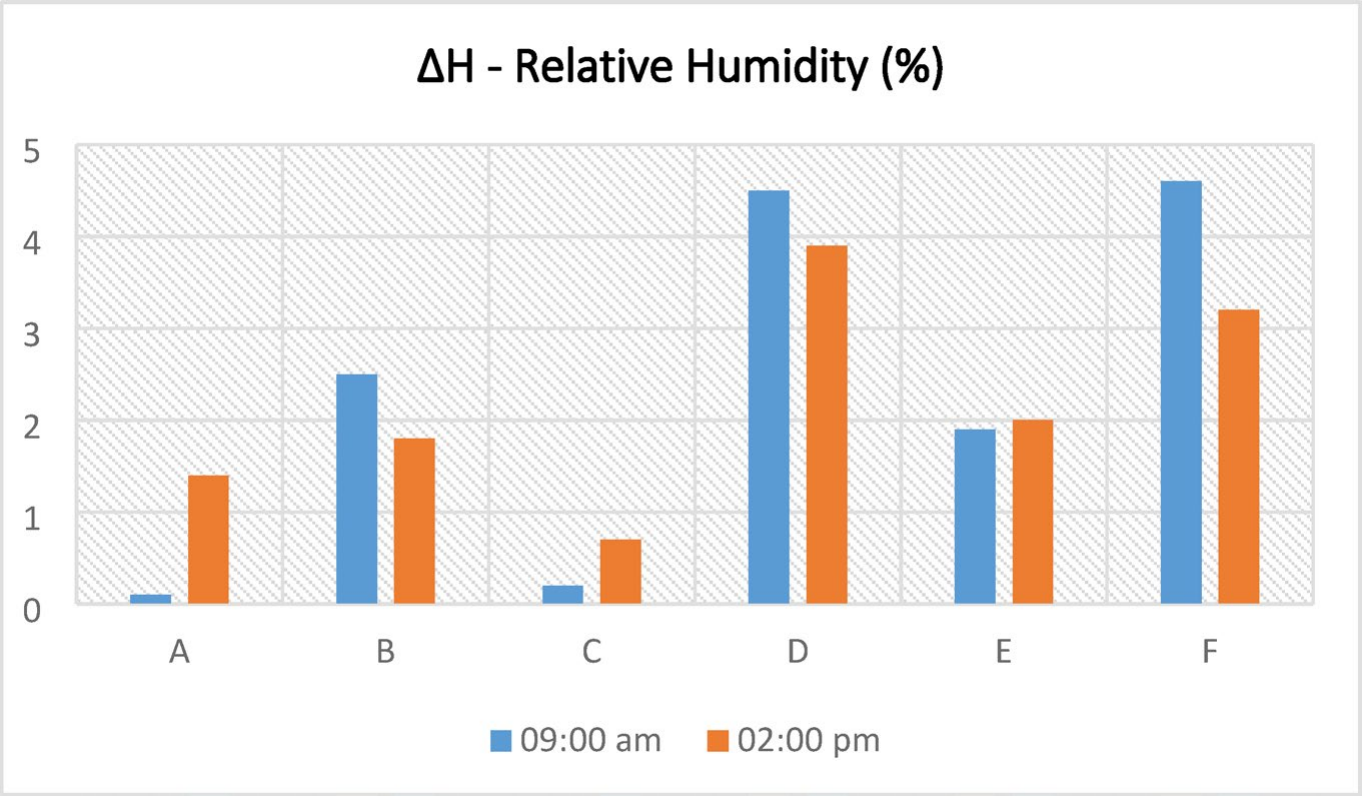
According to annual average values ΔH (H_max_−H_kabalti_).

Based on annual average relative humidity values, morning measurements showed a decrease in relative humidity from 46% (point B) to 38% (F3), indicating lower humidity at points with higher SVF. At noon, humidity values ranged between 36.6% (D) and 32.1% (F3) (Table 8). While humidity was lower in open areas, it was observed that kabaltıs and shaded areas maintained slightly higher humidity. These findings suggest that kabaltıs may serve as a protective buffer for the microclimate in terms of humidity (Fig. 11).

### Physiological equivalent temperature (PET)

**Fig. 12:**
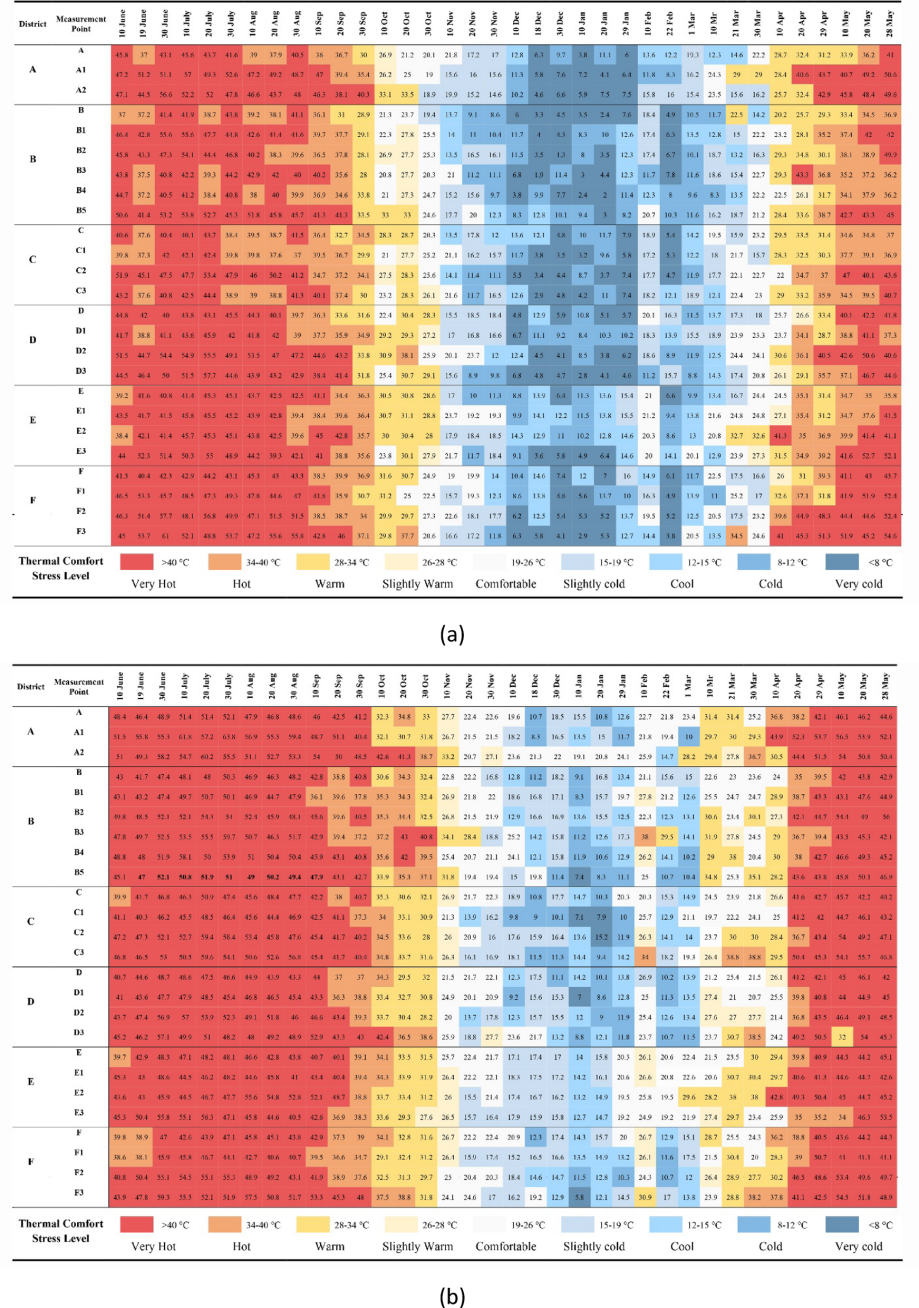
PET results calculated in (**a**) the morning and (**b**) the afternoon.

PET values calculated according to the measurements taken in the morning and noon hours were evaluated between the points within each region. The computed PET values morning results are shown in Fig. 12a and afternoon results are shown in Fig. 12b.

In the calculations with the measurements taken in the morning in the A region, the A1 point has the highest PET value with a value of 57 °C, the A point has the lowest PET value with a value of 3.8 °C (Fig. 12a), and in the afternoon, the A1 point has the highest PET value with a value of 63.8 °C and lowest PET value with a value of 8.3 °C (Fig. 12b). Points A1 and A2 were more exposed to very hot and very cold thermal stress both in the morning and in the afternoon compared to point A with an SVF value of 0. In the morning measurements, the number of comfortable days at point A is higher than at other points. However, the number of comfortable days at point A2 (SVF: 0.63) in the afternoon was higher, especially in winter. This situation is related to the fact that the SVF value of the A2 point is higher than the other points, and it receives more solar radiation. In the B region, in the morning, the highest PET value at the B1 point with 55.6 °C and the lowest PET value at the B2 point with 1.3 °C measured (Fig. 12a). In the afternoon, the highest PET value at the B3 point with 59.7 °C and the lowest PET value at the B5 point with 7.4 °C measured (Fig. 12b). The B5 point usually has a higher PET value up to about 15 °C in the morning compared to the other points. The fact that the B5 point has a very high SVF value and receives a large amount of solar radiation is a factor in this situation. Points B4 and B5 with higher SVF values in the afternoon, were exposed to hot and cold thermal stress compared to other points. In zone B generally, October and March in the morning and November and March in the afternoon are more comfortable. In the C region, in the morning, the highest PET value at the C2 point with 53.4 °C and the lowest PET value at the C3 point with 2.9 °C measured (Fig. 12a). In the afternoon, the highest PET value at the C3 point with 59.6 °C and the lowest PET value at the C1 point with 7.1 °C measured (Fig. 12b). In the morning measurements of point C2, it is seen that it is exposed to very hot and very cold thermal stress more than other points. According to PET values, the number of comfortable days of the C and C1 points in the afternoon is higher than the C2 and C3 points. Although the SVF value of the C1 point is greater than the C3, it can be said that the C1 point being more comfortable in some hours is due to the construction around it. The fact that the structure on one side of point C1 is in a semi-demolished state and is not a full mass, and the presence of a courtyard on the other side, has affected the PET value by making it possible to change the air movement at this point. In the D region, in the morning, the highest PET value at the D3 point with 57.41 °C and the lowest PET value at the D1 point with 7 °C measured (Fig. 12a). In the afternoon, the highest PET value at the D3 point with 57.7 °C and the lowest PET value at the D3 point with 2.8 °C measured (Fig. 12b). It was observed that there were differences in the PET results of the D1, D2, and D3 points with the same SVF value. In addition, the D1 and D3 points located on the same oriented streets are in the thermal stress range of very hot in the afternoon in the hot period of the year, and the difference between the PET values of these two points was generally 2–10 °C. This difference is related to the height of the surrounding buildings and street geometry. In the E region, the E3 point with the highest at 55 °C and the lowest at 3.6 °C in the morning (Fig. 12a), the E3 point with the highest at 56.3 °C and the lowest at 12.7 °C in the afternoon received its PET value (Fig. 12b). It was found that the number of comfortable days was at the highest point at E1 (SVF:0) in the morning measurements and at the most at E (SVF:0) in the afternoon measurements. It has been observed that point E2 is in the less comfortable range compared to other points, depending on the environmental construction features. In the F region, the F3 point with the highest at 61 °C and the lowest at 2.9 °C in the morning (Fig. 12a), the F3 point with the highest at 59 °C and the lowest at 5.8 °C in the afternoon received its PET value (Fig. 12b). It was observed that point F with a value of 0 SVF has more comfortable days than other points. It is seen that point F3 is exposed to very hot and very cold thermal stress more than other points during the morning and afternoon measurement hours. This situation is related to the amount of solar radiation affecting the F and F3 points. Urban geometry (building heights and street widths) affects the temperature in proportion to the amount of sunlight coming into the street texture, while the direction of the street and the spacing of the buildings affect the wind speed. In this case, the climate values in the street differ and affect the thermal comfort of people. The study conducted in Suriçi showed that SVF and PET affect each other but are not completely interdependent parameters. Some studies have shown that urban geometry and SVF value have effects on microclimate and human thermal comfort^8,12,26,27,30,36^. It was determined that changes such as street direction, building heights, and building spacings in urban design have a positive effect on thermal comfort^13,29,49,50^.

**Fig. 13:**
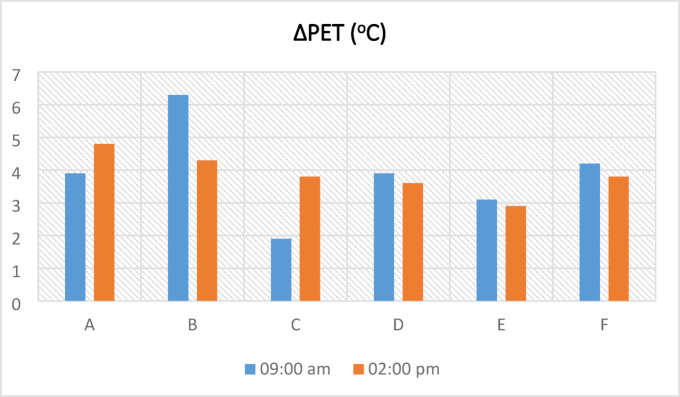
According to annual average values ΔPET (PET_max_ − PET_kabalti_).

The analysis of ΔPET values, derived from the annual average PET data in Table 9 and illustrated in Fig. 13, demonstrated the impact of kabaltıs on the thermal environment. Across all regions, the difference between the PET value under the kabaltıs in the morning and the peak PET value ranged from 1–7 °C, while at noon this difference remained between 2–5 °C, as clearly shown in Fig. 13. These findings, which are consistent with the detailed values presented in Table 9, indicate that kabaltıs provide a cooling effect, particularly compared to areas exposed to solar radiation. With increasing Sky View Factor (SVF), PET values generally tend to rise, and higher thermal stress is observed in open streets. Moreover, the results confirm that kabaltıs significantly enhance human thermal comfort by reducing PET values by an average of 4 °C (Table 9). Overall, these outcomes demonstrate that kabaltıs are an important and functional element in climatic design.

**Table 9:** Annual average PET values taken from the kabalti points (in ^o^C).

Time	Points					
	A	A1	A2			
09:00 a.m	26.3	30.7	30.2			
02:00 p.m	34.5	37.7	39.3			

Time	Points					
	B	B1	B2	B3	B4	B5
09:00 a.m	23.3	27.2	26.9	25.9	24.4	29.9
02:00 p.m	31.4	33	34.6	35.7	34.8	34.1

Time	Points					
	C	C1	C2	C3		
09:00 a.m	26.1	25.6	28	26.7		
02:00 p.m	32.1	30.4	34.3	35.9		

Time	Points					
	D	D1	D2	D3		
09:00 a.m	26.9	27.6	30.8	27.5		
02:00 p.m	31.3	31.1	32.9	34.9		

Time	Points					
	E	E1	E2	E3		
09:00 a.m	27.2	29.3	30.2	30.3		
02:00 p.m	32.9	33.3	35.8	32.7		

Time	Points					
	F	F1	F2	F3		
09:00 a.m	28.3	29.3	31.6	32.5		
02:00 p.m	32.3	30.6	33.8	36.1

The study results indicate that solar radiation is the most dominant factor affecting differences in PET values (Table 10). Points with high SVF values (e.g., B5, F3) showed PET values rising up to 60 °C in summer, while in shaded kabaltı areas, these values remained lower. This suggests that the primary cooling effect of kabaltıs stems from reducing direct solar radiation. However, differences in PET cannot be explained solely by SVF; in some areas (e.g., C2), low wind speed increased PET, whereas in others (e.g., A1), both orientation and SVF were effective. Points with the same SVF values but different PET outcomes (e.g., D1, D2, D3) indicate that environmental structures and street orientation influence PET by altering airflow and the distribution of solar radiation. Furthermore, the generally higher relative humidity in the morning may act as a secondary factor slightly reducing PET values.

**Table 10:** Dominant factors affecting PET values (the hottest measurement day of the year was July 20, 2020).

	SVF	PET		Dominant factor				
				Radiation (T_mrt_)	Wind	Canyon geometry		
		Morning	Midday			Orientation	Surrounding buildings	Shading
A	0	43.7	51.4					**√**
A1	0.40	49.3	57.2	**√**		**√**		
A2	0.63	52	60.2	**√**				
B	0	38.7	48					**√**
B1	0.21	47.7	50.7	**√**		**√**	**√**	
B2	0.26	44.4	54.3	**√**			**√**	
B3	0.26	39.3	55.5	**√**		**√**		
B4	0.45	38.4	50	**√**	**√**	**√**		
B5	0.96	52.7	51.9	**√**			**√**	
C	0	43.7	50.9					**√**
C1	0.40	42.4	48.5	**√**	**√**			
C2	0.27	53.4	59.4	**√**		**√**		
C3	0.18	44.4	59.6	**√**		**√**		
D	0	43.1	47.5					**√**
D1	0.47	45.9	48.5	**√**	**√**			
D2	0.47	55.5	53.9	**√**				
D3	0.47	57.7	51	**√**				
E	0	45.3	48.2					**√**
E1	0	45.5	46.2					**√**
E2	0.27	45.3	46.7	**√**		**√**		**√**
E3	0.20	55	56.3	**√**		**√**		
F	0	44.2	43.9					**√**
F1	0.14	47.3	46.7	**√**				
F2	0.50	56.8	55.1	**√**				
F3	0.67	48.8	52.1	**√**				

The findings indicate that the microclimate is influenced by these parameters. Low SVF values and shading reduce direct solar radiation, lowering ambient temperatures and preventing overheating in summer. Kabaltıs and low SVF provide sun protection, thereby helping to moderate temperature fluctuations. Narrow openings partially reduce wind speed but create a cooler and more comfortable environment compared to direct sunlight; this effect is further enhanced by directing airflow. The combined effects of these parameters show that kabaltıs in hot and dry climates like Diyarbakır are not simply structures that provide shade; they are also complex microclimatic regulators that direct wind flow, moderate temperature fluctuations, and influence humidity conditions. Therefore, kabaltıs can be considered an important element for climate-responsive design in hot and dry climates.

## Conclusions

The temperature results indicated that points with higher Sky View Factor (SVF) values exhibited higher temperatures than those with kabalti structures (A, B, C, D, E, E1, F). However, it was observed that air temperature measurements at locations with the same SVF value varied. This variation is influenced by factors such as the orientation of the measurement points, urban geometry, and wind effects. Morning and afternoon temperature measurements were different depending on the sun exposure of the areas where the points were located. It was found that the temperature differences between morning and afternoon were minimal at kabalti points, as these areas were not exposed to direct sunlight. Physiological Equivalent Temperature (PET) values measured at noon were generally higher than those recorded in the morning. The fact that PET values were not necessarily the highest or lowest on the dates when air temperatures peaked or dropped indicates that wind speed and humidity levels significantly influence PET. Although SVF and PET are related parameters, they are not fully interdependent. This is evidenced by variations in PET values between streets with similar SVF values. In such cases, differences in PET values are attributed to factors affecting wind speed—such as urban geometry (building heights and street widths), solar exposure within the street fabric, street orientation, and building spacing (e.g., at points C1–C3 and D1–D3). Morning PET results showed that most locations fell within the “very hot” and “hot” thermal perception ranges between May and September, the “comfortable” and “warm” ranges between March and October, and the “very cold” and “cold” ranges between December and January. Afternoon PET values indicated that the period from April to September generally fell within the “very hot” thermal perception range. October was mostly in the “hot” to “warm” range, while November, February, and March typically ranged from “comfortable” to “warm.” December and January were predominantly in the “cold” to “warm” range. Overall, it was determined that PET values measured in the morning were slightly lower than those in the afternoon. Additionally, the thermal perception during the summer period was generally classified as “very hot.” As modifying this condition in existing settlements is challenging, it is recommended that future urban designs take into account the effects of urban geometry on thermal comfort. The study also revealed that both temperature and PET values in shaded areas and under kabalti structures were slightly lower than in other locations. This shows that the kabalti is an advantageous use for regions with hot and dry climates. In Diyarbakir, a city with a hot and dry climate, the creation of shaded areas plays a significant role in protecting urban spaces from solar radiation and in lowering summer temperatures. Therefore, it is recommended to implement landscaping designs that provide shade during the summer while allowing solar access in the winter, particularly on streets where solar exposure is intense. Water features can also positively impact the microclimate by increasing ambient humidity. Accordingly, activating the use of pools in courtyard houses during the summer can contribute to higher humidity levels in the settlement. It is advised to use water elements in courtyards during hot periods to help create a favorable microclimate and improve thermal comfort conditions. In urban planning for hot and dry climates, it is essential to design environments that enable the controlled use of wind and incorporate shaded areas to enhance comfort. Moreover, in existing urban areas, introducing water features and thoughtful landscaping can improve environmental cooling during hot periods. These findings are supported by^51^ who showed that street geometry, orientation and shading regulate thermal stress at the city scale, while^52^ developed a method to estimate thermal stress and variation in streets at the city scale, thus assessing urban overheating beyond classical temperature measurements and highlighting the effects of shading, wind, radiation and humidity on outdoor thermal comfort. The study, based on temperature and PET measurements of streets in Kabaaltı and its surroundings, did not fully isolate many factors such as street orientation, urban geometry, and shade arrangements. This limitation is consistent with other measurement-based studies, such as^27^, who found that sunny open spaces were more comfortable in winter and shaded or semi-open spaces were more comfortable in summer in Erzurum. Similarly, Ahmadi Venhari et al.^53^ showed that SVF and the types of greenery and arrangement of urban spaces significantly affected thermal comfort in summer. Furthermore, the study highlighted the impact of different street orientations on PET values in Isfahan. Shalaby et al.^29^ indicated that H/W ratios and street orientation were important for improving daytime thermal comfort and reducing the urban heat island at night in Cairo, demonstrating that urban design parameters significantly affect human thermal comfort.

It is recommended that future research utilize field measurements, as well CFD-based modeling and simulation tools such as Rayman, SOLWEIG, ANSYS Fluent, and ENVI-met for more comprehensive analysis. Furthermore, investigating the applicability of architectural elements found in traditional urban fabrics like Kabaaltı/Sabat/Abbara could serve as a guide for climate-adapted urban design, particularly in hot and dry climates. It could also contribute to validating the effects of street orientation, shading, and greenery on human thermal comfort in different urban contexts.

## Data Availability

The datasets generated and/or analyzed during the current study are available from the corresponding author on reasonable request.
